# Antimicrobial resistance and virulence of *Pseudomonas* spp. among healthy animals: concern about exolysin ExlA detection

**DOI:** 10.1038/s41598-020-68575-1

**Published:** 2020-07-15

**Authors:** Lidia Ruiz-Roldán, Beatriz Rojo-Bezares, María de Toro, María López, Paula Toledano, Carmen Lozano, Gabriela Chichón, Lydia Alvarez-Erviti, Carmen Torres, Yolanda Sáenz

**Affiliations:** 1 Área de Microbiología Molecular, Centro de Investigación Biomédica de La Rioja (CIBIR), C/Piqueras 98, 3ªplanta, 26006 Logroño, Spain; 2 Plataforma de Genómica y Bioinformática, Centro de Investigación Biomédica de La Rioja (CIBIR), C/Piqueras 98, 26006 Logroño, Spain; 3 Área de Neurobiología Molecular, Centro de Investigación Biomédica de La Rioja (CIBIR), C/Piqueras 98, 26006 Logroño, Spain; 4 Área de Bioquímica y Biología Molecular, Complejo Científico-Tecnológico, Universidad de La Rioja, C/Madre de Dios 51, 26006 Logroño, Spain; 5 Present Address: Área de Bioquímica Y Biología Molecular, Complejo Científico-Tecnológico, Universidad de La Rioja, C/Madre de Dios 51, 26006 Logroño, Spain

**Keywords:** Bacterial pathogenesis, Bacterial genes

## Abstract

*Pseudomonas* is a ubiquitous genus that also causes human, animal and plant diseases. Most studies have focused on clinical *P. aeruginosa* strains from humans, but they are scarce on animal strains. This study was aimed to determine the occurrence of *Pseudomonas* spp. among faecal samples of healthy animals, and to analyse their antimicrobial resistance, and pathogenicity. Among 704 animal faecal samples analysed, 133 *Pseudomonas* spp. isolates (23 species) were recovered from 46 samples (6.5%), and classified in 75 different PFGE patterns. Low antimicrobial resistance levels were found, being the highest to aztreonam (50.3%). Five sequence-types (ST1648, ST1711, ST2096, ST2194, ST2252), two serotypes (O:3, O:6), and three virulotypes (analysing 15 virulence and quorum-sensing genes) were observed among the 9 *P. aeruginosa* strains. Type-3-Secretion System genes were absent in the six O:3-serotype strains that additionally showed high cytotoxicity and produced higher biofilm biomass, phenazine pigments and motility than PAO1 control strain. In these six strains, the *exlAB* locus, and other virulence genotypes (e.g. RGP69 pathogenicity island) exclusive of PA7 outliers were detected by whole genome sequencing. This is the first description of the presence of the ExlA exolysin in *P. aeruginosa* from healthy animals, highlighting their pathological importance.

## Introduction

*Pseudomonas* is a non-fermenting Gram-negative bacterium that colonizes different terrestrial and aquatic niches. Members of this genus inhabit in a wide variety of environments, due to their metabolic capacity and broad potential for adaptation to different conditions^1^. Moreover, various animals, such as birds or micromammals, have the ability to act as reservoirs of bacterial pathogens able to cause disease in humans, or environmental contaminations^2^. There is great interest in *Pseudomonas* because of its involvement in plant and human diseases, but also by their potential in biotechnological applications^3^.

This genus comprises a wide variety of species, including the opportunistic pathogen *P. aeruginosa* which is of increasing medical and veterinary importance, causing infections mostly in patients with compromised immune systems, or in people suffering from cystic fibrosis^1^. It is also a cause of diseases in livestock and companion animals, including urinary tract infections in dogs, mastitis in dairy cows, and endometritis in horses^4,5^.

*P. aeruginosa* has intrinsic resistance to several antibiotics and capacity to acquire new resistance mechanisms. Acquired resistance in *P. aeruginosa* is multifactorial and attributable to chromosomal mutations and to the acquisition of genes by horizontal transfer^6^. Integrons are genetic elements that play an important role in antimicrobial resistance transfer. These elements are able to capture, integrate, and express gene cassettes encoding proteins associated with antimicrobial resistance. Furthermore, these integrons could be mobilised by plasmids or transposons^7^.

Nowadays, most of the antipseudomonal agents are reserved for human medicine to preserve their activity, and the treatment of infected animals only relies on a few antibiotics. Nevertheless, strains resistant to these agents are appearing in animal samples^8^.

The pathogenicity of *P. aeruginosa* strains is associated with different virulence factors, that include proteases, elastases, exotoxins, or phenazine pigments, some of which are under the control of a cell density recognition mechanism called quorum sensing (QS)^9^. The major virulence determinants of this microorganism are the Type 3 Secretion System (T3SS) and its toxins, termed effectors (ExoU, ExoS, ExoT, ExoY). T3SS is a molecular syringe that delivers virulence effectors directly into the cytoplasm of infected host cells^1^. PAO1 and PA14 classical clonal groups possess these T3SS machinery and toxins, but they are absent in PA7 clonal group, classified as a taxonomic outlier^10,11^. On the other hand, the PA7 strains harbour a novel two partner secretion (TPS) system, called exolysin ExlA^11^. The ExlA toxin, together with its cognate porin (ExlB), exhibits high cytotoxicity activity in host cells^12^.

Several studies have demonstrated that clinical and environmental isolates show common genotypic and phenotypic properties. However, the acquisition of resistance mechanisms is more frequently observed among clinical isolates than among environmental ones^13^. Despite the idea that *P. aeruginosa* grows in different niches, the majority of studies have focused on clinical *P. aeruginosa* strains from humans, but much less on animal and environmental strains^2,14^.

The aim of this study was to determine the occurrence of *Pseudomonas* spp. isolates among faecal samples of healthy animals, and to analyse their characteristics including antimicrobial resistance, virulence factors, and molecular typing.

## Results

### Isolates of *Pseudomonas* spp.

A total of 133 *Pseudomonas* spp. were isolated from 46 animal samples (6.5%) as follows (number of isolates): wild boars (57), mallards (30), ticks (17), farm animals (9), pets (10), rabbits (5), micromammals (3), and deer (2) (Supplementary Table S1). These isolates were classified into the following species (number of isolates): *P. aeruginosa* (33), *P. putida* (23), *P. fluorescens* (17), *Pseudomonas* spp. (12), *P. gessardii* (7), *P. fragi* (6), *P. psychrophila* (6), *P. koreensis* (5), *P. fulva* (4), *P. protegens* (3), *P. mendocina* (2), *P. reactans* (2), *P. lundensis* (2), *P. monteilii* (2), *P. plecoglossicida* (1), *P. pseudoalcaligenes* (1), *P. baetica* (1), *P. brassicacearum* (1), *P. cedrina* (1), *P. corrugata* (1), *P. synxantha* (1), *P. viridiflava* (1), and *P. trivialis* (1). The thirty-three *P. aeruginosa* isolates were recovered from five samples of wild boars and one of sheep. An additional table file shows all this information in more detail (Supplementary Table S2).

### Antimicrobial susceptibility testing

One hundred thirty-three *Pseudomonas* spp. isolates were screened for the antimicrobial susceptibility testing, and they showed resistance to (percentage of resistance): aztreonam (50.4%), meropenem (12%), doripenem (11.3%), cefepime (8.3%), ceftazidime (2.2%), piperacillin–tazobactam (0.7%), and imipenem (0.7%). 46.6% of the isolates were susceptible to all antipseudomonal agents tested; but on the other hand, eight isolates showed a multidrug resistance phenotype (Supplementary Table S2). No isolate showed class A carbapenemase, extended-spectrum beta-lactamase (ESBL), or metallo-beta-lactamase (MBL) phenotypes.

### Molecular typing

Pulsed-field gel electrophoresis (PFGE) patterns were analysed by species. Seventy-five different PFGE patterns were detected among the 133 *Pseudomonas* spp. isolates (Supplementary Table S2). One strain with different PFGE pattern and species per sample was included in this study. Additionally, three pairs of isolates from three samples were included because they showed the same PFGE profiles but different resistance phenotypes, i.e., two *P. fluorescens* from a tick sample (Ps689 and Ps693), two *P. aeruginosa* from a sheep (Ps531 and Ps533), and other two *P. aeruginosa* from a wild boar (Ps633 and Ps634). Moreover, two *P. aeruginosa* isolates from the same wild boar sample showed different PFGE patterns, thus both strains (Ps620 and Ps624) were included in the study. Indistinguishable patterns were detected only among isolates from the same sample (Supplementary Table S2), selecting one strain for further studies; with the exception of 19 *P. aeruginosa* isolates that were recovered from three different wild boar samples (9, 7 and 3 isolates, respectively) which showed the same PFGE pattern, and one strain from each sample and antimicrobial phenotype was selected (Ps616, Ps624, Ps633, and Ps634).

A total of 80 *Pseudomonas* strains were finally included in this study for further characterization, and 9 of them were *P. aeruginosa* (Table 1).

**Table 1:** Characteristics of the studied *P. aeruginosa* strains recovered from animal faecal samples.

Strain	Origin	Serotype	PFGE	MLST	Resistance phenotype^a^	Virulence profile^b^	Biofilm (%)^c^		Phenazines (%)^c^		Elastase (%)^c^	Motility (mm^2^)	
							CV	FDA	Pyocyanin	Pyorubin		Swimming	Swarming
Ps531^d^	Sheep	O:3	1	ST2096^**g**^	Susceptible	III	350.1	92.9	130.2	83.8	87.8	6,300.6	6,400.0
Ps533^d^	Sheep	O:3	1	ST2096^**g**^	ATM, MEM	III	393.6	95.8	130.4	130.9	75.2	6,320.2	6,400.0
Ps616	Wild boar	O:3	40	ST1711	Susceptible	II	438.6	104.1	121.1	120.8	105.7	6,400.0	6,400.0
Ps624^e^	Wild boar	O:3	40	ST1711	Susceptible	II	306.7	86.1	240.6	253.6	110.1	6,400.0	6,400.0
Ps633^f^	Wild boar	O:3	40	ST1711	Susceptible	II	292.3	90.0	323.0	235.8	68.4	6,400.0	6,400.0
Ps634^f^	Wild boar	O:3	40	ST1711	DOR	II	338.8	88.5	198.5	188.5	191.9	6,400.0	6,400.0
Ps631	Wild boar	O:6	42	ST1648	Susceptible	I	166.1	99.8	45.4	87.3	98.3	6,400.0	6,400.0
Ps620^e^	Wild boar	O:6	41	ST2252^**g**^	Susceptible	I	176.5	128.9	37.7	41.2	146.7	6,400.0	6,400.0
Ps638	Wild boar	O:6	43	ST2194^**g**^	Susceptible	I	146.2	141.5	37.6	44.9	100.5	5,117.6	6,400.0

^a^Susceptible: this strain was susceptible to all 13 antibiotics tested; *ATM* aztreonam, *MEM* meropenem, *DOR* doripenem.

^b^Virulotype I: *exoS, exoY, exoT, exoA, lasA, lasB, aprA, rhlAB, rhlC, rhlI, rhlR, lasI, lasR. *Virulotype II*: exlA, exoA, lasA, lasB, aprA, rhlAB, rhlI, rhlR, lasI, lasR. *Virulotype III*: exlA, lasA, lasB, aprA, rhlAB, rhlI, rhlR, lasI, lasR.*

^c^Biofilm biomass production (CV), metabolic activity of bacteria within biofilm (FDA), pyocyanin and pyorubin production, and elastase assay showed as percentage compared to control *P. aeruginosa* PAO1 strain values.

^d^Both strains were recovered from the same sheep sample.

^e^Both strains were recovered from the same wild boar sample.

^f^Both strains were recovered from the same wild boar sample.

^g^New allelic combinations.

The sequence types (ST) were determined among these nine *P. aeruginosa* strains using the multilocus sequence typing (MLST) method. Five different sequence types were observed (Table 1). Three ST were firstly described in this study, and registered in the MLST database as ST2096, ST2194 and ST2252. Five *P. aeruginosa* strains, isolated from three wild boars, were ascribed to ST1711 (4 strains) and ST1648 (the remaining one).

### Serotyping

Two different serotypes were identified among the 9 *P. aeruginosa* strains. Six of them (Ps531, Ps533, Ps616, Ps624, Ps633, and Ps634) showed serotype O:3, while the remaining strains (Ps620, Ps631, and Ps638) were serotype O:6 (Table 1).

### Detection and characterization of integrons

Class 1 integron was detected in 5 out of 80 isolates studied (6.2%), whereas no isolate showed class 2 or 3 integrons. *Pseudomonas* sp. Ps656, *P. fluorescens* Ps658 and *P. fragi* Ps662 isolated from wild boar samples, and *P. fluorescens* Ps689 and Ps693 recovered from ticks, were the integron-positive isolates. No *P. aeruginosa* strains harboured integron structures (Supplementary Table S2). The usual class 1 integron structures are characterised by the presence of the *intI1* gene, a gene cassette promoter (Pc), the 3′-conserved segment (3′-CS) (*qac*Δ*E* + *sul1* genes) and the gene cassettes included in their variable region. These integrons were analysed in the 5 positive strains.

A new class 1 arrangement, regulated by a weak promoter (PcW) and an active P_2_ promoter, was detected in *Pseudomonas* sp. Ps656. Its variable region was composed by a gene with unknown function, *gcuE28* and an aminoglycoside adenylyltransferase gene (*aadA1a*). This new class 1 integron was submitted to INTEGRALL (https://integrall.bio.ua.pt/) and GenBank databases, and the name In1180 and the accession number KT368820 were assigned.

*P. fluorescens* Ps658 and *P. fragi* Ps662 harboured empty integrons (absence of gene cassettes inside their variable regions) and with the complete *qacE* gene without the *sul1* gene at the 3′-CS. Class 1 integron of Ps658 was regulated by a hybrid promoter (PcH1), and class 1 integron of Ps662 showed a weak promoter (PcW). Additionally, the class 1 integron of *P. fragi* Ps662 possessed typical features of a Tn*402-*like class 1 integron, carrying the *tni* transposition module *tniC, tniQ, tniB*, but lacking the *tniA* gene.

### Detection of genetic virulence markers

The distribution of 15 genes involved in virulence, T3SS and QS system was investigated among the 9 *P. aeruginosa* strains, and three different virulotypes were obtained (Table 1).

All strains amplified *lasA*, *lasB*, *aprA*, and *rhlAB* genes, as well as the genes involved in QS system (*lasI, lasR, rhlI* and *rhlR*). However, no strains showed *exoU* gene, and only the six wild boar strains (virulotypes I and II) amplified *exoA* gene. The T3SS *exoS*, *exoY* and *exoT* genes and the *rhlC* gene were detected in three *P. aeruginosa* strains (virulotype I), but they were absent among the remaining six strains (virulotypes II and III). The *exlA* gene amplified in all these six T3SS-negative strains.

### Biofilm quantification

**Figure 1:**
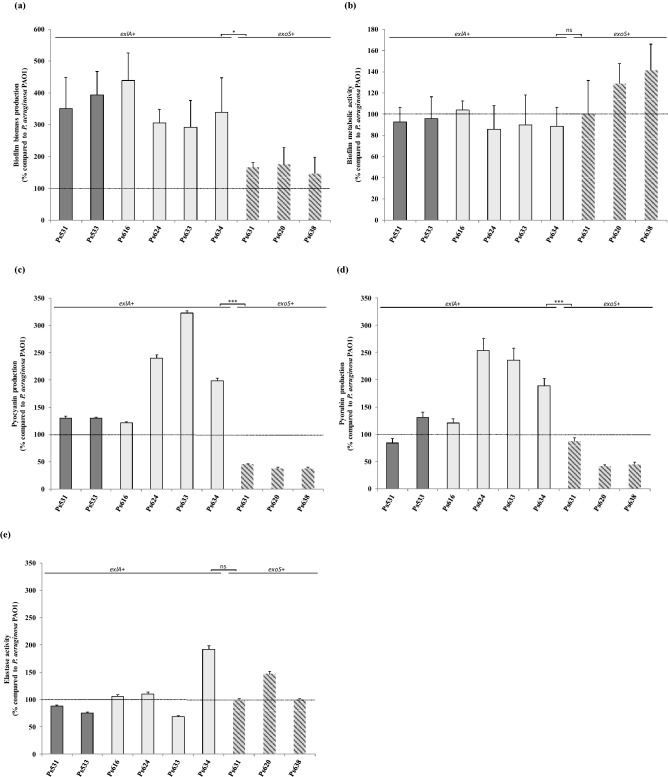
Phenotypic assays of virulence of the 9 *P. aeruginosa* strains. (**a**) Biofilm biomass production determined by staining with crystal violet (CV); (**b**) metabolic activity within biofilm determined by staining with fluorescein diacetate (FDA); (**c**) Pyocyanin production assay; (**d**) Pyorubin production assay, and (**e**) Elastase assay. Dotted line (PAO1 value = 100%). Data expressed as mean ± SD. Virulotype III-strains (dark grey bar); virulotype II-strains (light grey bar); virulotype I-strains (grey striped bar). Two-tailed *t* test *p < 0.05, ***p < 0.001, *ns *no significant differences.

The total biofilm biomass (CV) and the bacterial metabolic activity inside the biofilm structure (FDA) of the 9 *P. aeruginosa* strains were analysed in comparison to the control strain *P. aeruginosa* PAO1, and the detected percentages are shown in Table 1 and Fig. 1a,b. All strains produced more biofilm biomass than the control strain. The *exlA*-positive strains (serotype O:3 and virulotypes II and III) showed higher biofilm biomass production than *exoS*-positive strains (p = 0.031), and three to four times higher CV values than PAO1 strain (Fig. 1a). In FDA assay, 66.7% of strains had a lower metabolic activity than the control strain. There were no statistically significant differences between FDA values of *exlA*-positive and *exoS*-positive strains (p = 0.113) (Fig. 1b).

### Phenazine pigment and elastase production, and motility

Percentages of pyocyanin and pyorubin production; as well as elastase activity, relative to PAO1 strain levels, are summarized in Table 1 and Fig. 1c–e. All O:6-serotype (virulotype I) *P. aeruginosa* produced lower levels of both phenazines than the control strain. Higher pyocyanin values than PAO1 were observed among all *exlA*-positive *P. aeruginosa* strains (O:3-serotype, virulotypes II and III), and pyorubin production was higher than 120% in all but one of those strains. Significant differences (p < 0.001) in both phenazine productions were observed between *exlA*-positive and *exoS*-positive strains. Variable elastase activity (range 68.4–191.9%), and high swimming (area ranged from 5,117.6 to 6,400 mm^2^) and swarming (6,400 mm^2^) motility values were detected among the 9 *P. aeruginosa* strains (Table 1). There were no statistically significant differences in elastase production between *exlA*-positive and *exoS*-positive strains (p = 0.383) (Fig. 1e).

### Whole genome sequencing (WGS)

Three *exlA*-positive *P. aeruginosa* strains, Ps533, Ps616 and Ps633, were selected to analyse their virulence markers in depth by WGS.

The presence of the new TPS system, i.e. the operon *exlBA*, and the absence of the whole T3SS loci (five operons and 36 genes), the T3SS exotoxin genes (*exoS*, *exoU*, *exoY* and *exoT*) and *rhlC* gene were confirmed in all strains analysing their genomes. The operon *exlBA* was localised in the gap between PA0873 and PA0874 genes from *P. aeruginosa* PAO1, and at the same position as in *P. aeruginosa* PA7, i.e. between PSPA7_4640 and PSPA7_4643 (*phhR* gene) genes. Moreover, our strains showed the genes required for flagella and type IV pili formation (except *pilA* gene), for alginate biosynthesis, the *oprA* (91% identity), and the *txc* cluster, included in the RGP69 pathogenicity island exclusive of PA7. On the other hand, unlike described in PA7 strain, these strains showed not fused the *phzA1* and *phzB1* genes, responsible of the phenazine production. However, phenazine *phzH* gene and phospholipase D *pldA* gene were not detected either in our strains or in *P. aeruginosa* PA7, but were described in *P. aeruginosa* PAO1 and PA14 reference strains.

**Figure 2:**
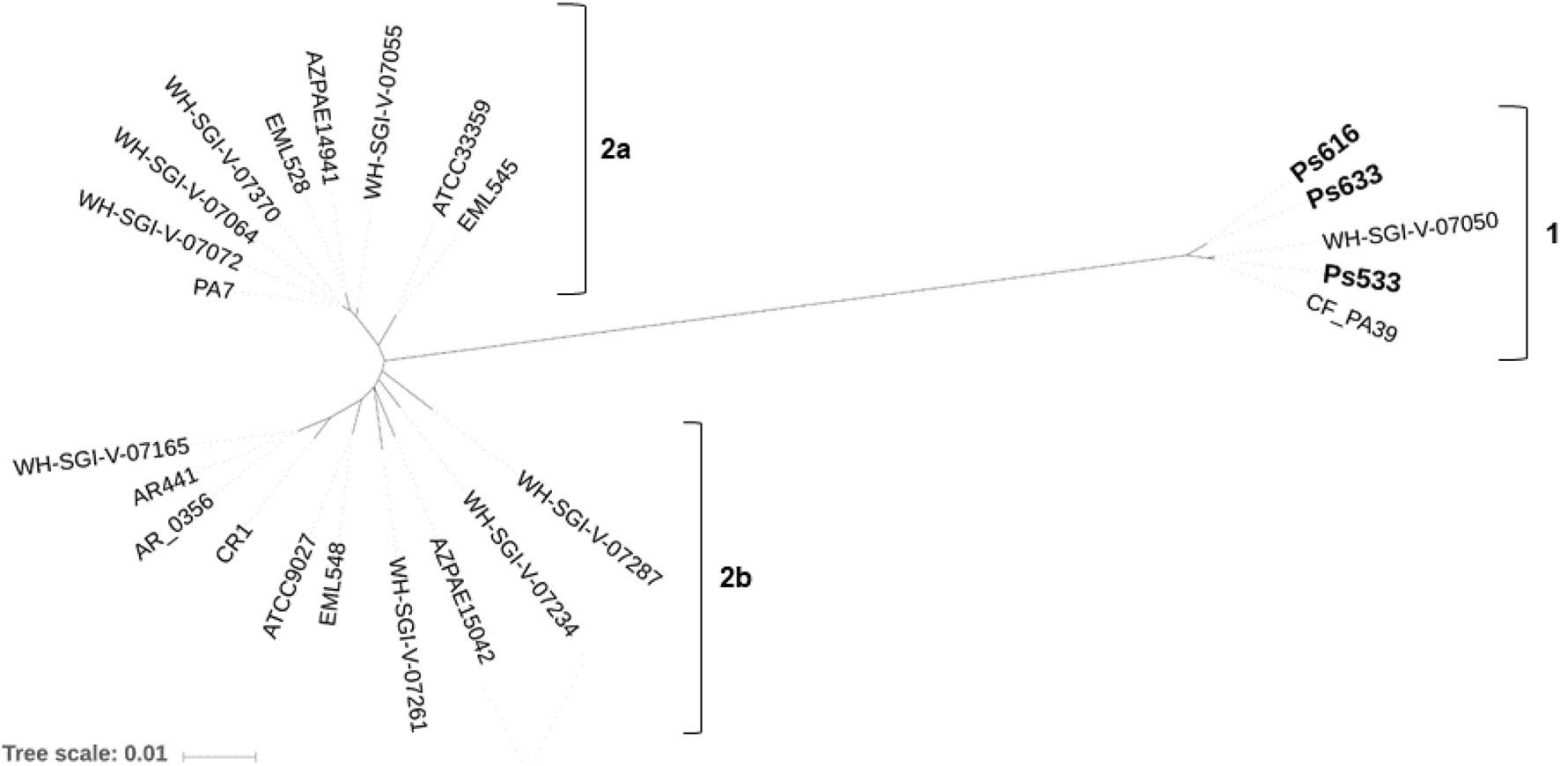
Phylogenetic tree of the *exlA*-positive sequenced *P. aeruginosa* strains. The dendrogram was constructed using a Neighbour-Joining algorithm, using ExlA amino acid sequence as an alignment. Bootstrap values for 10,000 replicates. *P. aeruginosa* of this study (Ps533, Ps616 and Ps633) are marked with bold letters. Amino acid sequences of the remaining *exlA*-positive strains were obtained from the *Pseudomonas* Genome Database^15^. Two clades (1 and 2) and subclades (2a and 2b) are highlighted with square brackets.

The phylogenetic tree (Fig. 2) shows the distance of the ExlA protein of our strains in comparison with *exlA*-positive *P. aeruginosa* strains that were previously described or published (*Pseudomonas* Genome Database^15^, date: 2019-02-05). These *P. aeruginosa* strains were divided in two main clades (1 and 2), whereas clade 2 was classified in two subclades (2a and 2b), reflecting the differences in ExlA amino acid substitutions. *P. aeruginosa* Ps533, Ps616 and Ps633 showed an important ExlA evolution regarding *P. aeruginosa* PA7 and the remaining *P. aeruginosa* reference strains. Indeed *P. aeruginosa* Ps533, Ps616 and Ps633 were grouped in the clade 1, the same clade as CF_PA39 strain, an isolate (serotype O:3, ST1744) collected from a sputum sample of a cystic fibrosis patient^16^.

The ExlA protein (1651 aa) of our strains showed 89 amino acid changes respect to the *P. aeruginosa* PA7 sequence, but only 11 amino acid substitutions comparing to *P. aeruginosa* CF_PA39 sequence (Supplementary Fig. S1 and Table S3). Moreover, *P. aeruginosa* Ps616 and Ps633 strains, both belonging to virulotype II, showed the same ExlA amino acid sequence, but 6 amino acid substitutions were detected in comparison with Ps533 strain. Nevertheless, no changes were detected in the conserved motifs involved in TPS secretion comparing with the PA7 or CF_PA39 ExlA proteins (Supplementary Fig. S1). Curiously, Ps533, Ps616, Ps633 and CF_PA39 strains showed one extra RGD (Arg-Gly-Asp) motif in their ExlA protein, in contrast to PA7 ExlA protein. These motifs are involved in cell-to-matrix interactions. On the other hand, the ExlB protein (570 aa) of our strains showed 17 and 5 amino acid changes respect to those of the PA7 and CF_PA39 strains, respectively (Supplementary Fig. S1 and Table S3). The ExlB sequences of Ps616 and Ps633 were identical, but showed three substitutions in comparison with Ps533 sequence.

### Strain cytotoxicity analysis

**Figure 3:**
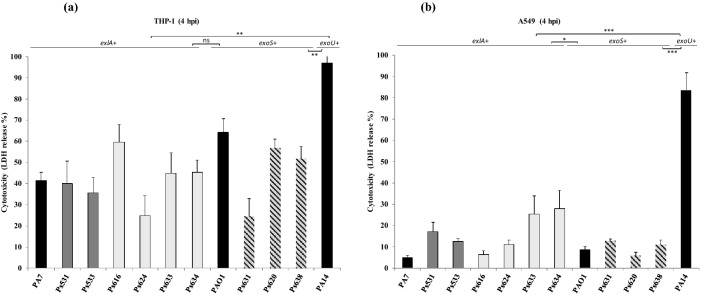
Cytotoxicity levels of *P. aeruginosa* strains studied by LDH release in THP-1 monocytes (**a**), and in A549 epithelial cells (**b**). Data expressed as mean ± SEM (n = 4). One-way Anova test *p < 0.05, **p < 0.01, ***p < 0.001, *ns *no significant differences. *P. aeruginosa* PA7 (*exlA*-positive), PAO1 (*exoS*-positive) and PA14 (*exoU*-positive) strains (black bars) were used as reference strains. For the A549 test, PAO1 is used as negative control, and PA14 as positive control. Virulotype III-strains (dark grey bar); virulotype II-strains (light grey bar); virulotype I-strains (grey striped bar).

The cytotoxicity of the 9 *P. aeruginosa* strains was analysed using the THP-1 monocyte and A549 epithelial cell lines. As Fig. 3 shows, *P. aeruginosa* PA14 reference strain (*exoU*-positive) showed the highest cytotoxicity level in both cell lines. *exlA*-positive *P. aeruginosa* strains showed a medium cytotoxicity potential (average 42%) similar to the cytotoxic effect of *exoS*-positive strains that killed 50% of the THP-1 cells (Fig. 3a). Low cytotoxicity was observed in A549 cells (Fig. 3b). Cell death values for *exlA*-positive *P. aeruginosa* strains (virulotypes II and III) ranged from 59.3 to 73.6%, which was at the same range as PA7 level (61.5%). On the other hand, strains belonging to virulotype I (*exoS*-positive), including *P. aeruginosa* PAO1 control strain, showed lower cytotoxicity values ranged from 43.2 to 60.2%. Statistical differences were showed among all the strains grouped according to *exoS*, *exoU* and *exlA* presence (P < 0.0001) (Fig. 3c).

## Discussion

Humans, animals, and environment are reservoirs of bacteria carriers of antimicrobial resistance and virulence genes that could be mobilized to human pathogens such as *P. aeruginosa*^2,17^. Animals are important reservoirs and potential disseminators of bacteria and their antimicrobial resistance genes, because of its proximity to humans^17^. However, the studies about antimicrobial resistance have mainly focused on companion animals, and those about virulence characterization are even absent in *P. aeruginosa* from animal origin.

In our study, *Pseudomonas* spp. isolates were detected in 46 faecal samples and ticks (6.5%), and the highest prevalence was detected among duck (93%) and wild boar samples (29%) that respectively were 28% and 41% regarding the 46 positive samples. A great variability of *Pseudomonas* species was observed, being *P. putida* and *P. aeruginosa* the most predominant ones. *P. aeruginosa* was detected among sheep and wild boar samples. This species is one of the most relevant opportunistic human pathogens, although other species such as *P. putida, P. fluorescens, P. mendocina*, *P. fulva* or *P. monteilii*, have also caused human clinical infections. Additionally, other plant and animal pathogenic species were also isolated in our study, such as *P. viridiflava*, *P. plecoglossicida* or *P. baetica*^18–20^.

Regarding antimicrobial susceptibility, all our *Pseudomonas* spp. recovered from healthy animals were susceptible to aminoglycosides, and fluoroquinolones, and low resistance rates were observed to carbapenems and cephalosporins. Neither MBL nor class A carbapenemase were detected, so other mechanisms such as AmpC hyperexpression, porin alterations or active efflux pumps could be involved in those carbapenem-resistance phenotypes. In contrast, a high resistance level to aztreonam was observed. The presence of these antimicrobial resistant *Pseudomonas* in healthy animals increases the risk for animal or human health, because these animal faecal wastes contaminate soils and aquifers, and contribute to the dissemination of antimicrobial resistant bacteria among human, animal and environmental origins.

Additionally, the horizontal dissemination of antimicrobial resistance genes has been previously associated with the presence of mobile or mobilizable genetic elements^21^. Indeed, the presence of integrons is high in clinical antimicrobial-resistant *P. aeruginosa* strains^22^. However, in our study, class 1 integron was only found in five of the 80 *Pseudomonas* spp. strains, but none of them were *P. aeruginosa* species. The new In1180 class 1 integron (GenBank KT368820) was firstly described in this work. Two strains showed empty gene cassette structures in their variable regions, one of them in a Tn*402-*like class 1 integron, carrying the *tni* transposition module. In the future and under environmental pressures, these structures could allow the adaptation of the bacteria by integrating antimicrobial resistance genes^7^. Tn*402-*like class 1 integrons are considered as the progenitors of the frequent multidrug resistance integrons. Several studies have added that clinical class 1 integrons lost the Tn*402* transposition functions, whereas those Tn*402-*like empty class 1 integrons seem to be more frequently recovered from environmental sources^23^. To our knowledge, this is the first time that this type of structure is described in healthy animal strains.

Our study highlights a high genetic diversity among *Pseudomonas* species; there is a non-clonal epidemic structure among the different animal origins or ecological niches. However, there are studies where *P. aeruginosa* exhibits an epidemic population structure^24,25^. “High-risk clones” associated with multidrug-resistant clinical *P. aeruginosa* strains were not identified among our strains that, on the other hand, showed three new STs (ST2096, ST2194, ST2252). ST1711, reported in this study among *P. aeruginosa* from wild boars, was firstly detected in *P. aeruginosa* from dairy cow respiratory infection in France^4^.

Analysing antimicrobial susceptibility among these 9 *P. aeruginosa*, the 78% were susceptible to all antipseudomonal agents tested, and only one strain was meropenem-resistant. These results contrast with the antimicrobial resistance percentages reported in human or animal clinical *P. aeruginosa* strains^4,22^.

*P. aeruginosa* uses its big arsenal of pathogenicity factors to interfere with host defences. This arsenal includes adhesins and secretion toxins, effector proteins, proteases, elastases and phenazine pigments that facilitate the adhesion and biofilm production; module or alter the cascades of the host cells and act against extracellular matrix^1,9^. The LuxI/LuxR QS type (LasI/LasR and RhlI/RhLR) genes are molecular communication systems among bacteria that, according to bacterial density, are able to express different cascades of genes, and regulate virulence and biofilm formation^9^. In our study, all *P. aeruginosa* strains possessed these QS system genes, including those responsible of the autoinducers biosynthesis (*lasI* and *rhlI*), conferring to *P. aeruginosa* a significant virulence phenotype. Moreover, scarce studies have analysed these mechanisms in non-clinical *P. aeruginosa* strains^26^, and our work is one of the first biofilm, phenazines and motility studies in animal strains.

Recently, whole-genome analysis assays have grouped environmental and clinical *P. aeruginosa* isolates into three major groups, called PAO1, PA14 and PA7, mainly differentiated by their cytotoxicity and virulence^27^. The majority of the worldwide analysed isolates have been classified between the two first groups that possess the T3SS machinery, i.e. ExoS (but not ExoU), ExoT and ExoY (*P. aeruginosa* PAO1), or ExoU (but not ExoS), ExoT and ExoY effectors (*P. aeruginosa* PA14). The third group (*P. aeruginosa* PA7), qualified as clonal outliers, included the T3SS-lacking strains that secreted the ExlA exolysin to cause host infections^27^. Curiously, the *P. aeruginosa* of our study were grouped in three virulotypes according to the analysis of fifteen genes involved in virulence, T3SS and QS systems. The QS system genes (*lasI*, *lasR*, *rhlI*, and *rhlR*) were amplified in all of our strains; as well as *lasA*, *lasB*, *aprA* and *rhlAB* genes. The ExoU toxin-effector was absent in our strains, as well as the remaining T3SS effectors (ExoS, ExoY, and ExoT) in the six strains with the serotype O:3. Nevertheless, *exlA* gene was amplified in these six T3SS-lacking strains. The locus *exlBA* was detected in their genome, as well as other virulence markers associated with *P. aeruginosa* PA7 clonal outlier^10,28^, such as the pathogenicity island RGP69 that includes the *txc* cluster, a Type 2 Secretion System (T2SS) exclusive of PA7 outlier group^11^; or the OprA porin, which is absent in PAO1 and PA14 clonal groups^10,11^. On the other hand, *P. aeruginosa* strains belonging to virulotype II amplified the *exoA* gene, encoding exotoxin A, which is infrequent among PA7 outlier group but detected in CF_PA39 strain^11,16^.

Interestingly, the origin of the PA7-related strains has not been restricted to country or continent, or to a single environment. Many *P. aeruginosa* strains belonging to PA7 clonal group have been isolated from different infections, as cystic fibrosis patients, urinary or peritoneal infections. However, other *P. aeruginosa* strains have been isolated from the environment, as the strains of our study. These features would suggest that the *exlA*-positive strains are able to colonize different ecological niches^12^. Other studies have divided *exlA*-positive strains into two different groups, one group (Group A) includes PA7 strain, thus can be designated as PA7-like strains; whereas the other group (Group B) is phylogenetically closed to PAO1/PA14 strains and significantly less toxic than Group A strains^12^. As observed in this study, the ExlA of our *P. aeruginosa* strains were grouped into a different cluster of PA7 reference strain, but into the same group as other PA7-like strains, Group B such as CF_PA39 strain, isolated from a patient with cystic fibrosis in Belgium^16^. Based on phylogenetic studies and confirming these results, the *exlA*-positive strains might have evolved from at least two different ancestors^12^.

Additionally, those six *exlA*-positive strains produced more biofilm biomass (more than three times), phenazines, and motility (swimming and swarming) than the control PAO1 strain. On the other hand, the three remaining T3SS-positive *P. aeruginosa* strains were serotyped as O:6, one of the predominant serotypes in clinical and non-clinical strains^29,30^, and showed more biofilm biomass (range 146–166%) and motility, but lower phenazine pigment production than the PAO1 strain.

As *P. aeruginosa* invades epithelial and phagocytic cells, the cytotoxicity of the strains was investigated in an epithelial cell line (A549) and a monocyte cell line (THP-1). Both cell lines were highly sensitive to most of the *exlA*-positive strains studied. The *exlA*-positive and PA7 strains showed higher cytotoxicity levels in A549 cell line than *exoS*-positive and PAO1 strains; whereas no significant difference was found among those strains in THP-1 cells. On the other hand, *P. aeruginosa* PA14 (*exoU*-positive) produced the highest cytotoxicity levels in both cell lines. In epithelial cells, ExoU or ExlA toxins trigger necrotic cell death, and ExoS toxin induces apoptosis, which involves that *exoU*-positive and *exlA*-positive strains were more virulent than *exoS*-positive ones, as previously reported^12,28,31^. In monocytes, ExoU induces a caspase-1 independent necrosis and ExlA and ExoS induce pyroptosis, the different mechanisms were related with the cytotoxic effect observed^32,33^. Toxicity is generally strain-dependent, and different factors could be involved, such as ExlA or ExoS secretion. Regarding *exlA*-positive strains, previous experiments performed by Reboud et al.^27^ showed that *P. aeruginosa* PA7 strain was more toxic than *P. aeruginosa* CF-PA39 strain, whereas our *exlA*-positive strains showed similar or even higher cytotoxic effect than PA7 strain. This is the first time that *exlA*-positive *P. aeruginosa* strains were described in healthy animals, highlighting their pathological importance.

## Conclusions

The genus *Pseudomonas* is widely distributed in healthy animals (wild, companion or farm animals). A variety of different *Pseudomonas* species, some also pathogenic to humans, such as *P. aeruginosa, P. putida*, or *P. fulva* have been detected in this study. Furthermore, there is a non-clonal epidemic structure among the different animal strains or ecological niches. The occurrence of *P. aeruginosa* in faecal samples of animals (0.8%) was lower than in hospitals and human infections, and in healthy human faecal samples. In addition, low antimicrobial resistance levels were found in these isolates in comparison with clinical strains. However, under antibiotic pressure, integrons or other mobilizable elements could constitute an effective way of spreading multiple antibiotic resistances between the different environments. Regarding *P. aeruginosa* virulence, this is the first description of the presence of the ExlA exolysin in *P. aeruginosa* from healthy animals, highlighting the pathological importance of these strains.

Antimicrobial resistance and virulence should be closely monitored in animal *P. aeruginosa* isolates in the future, in line with possible transferences or disseminations among different ecological niches.

## Methods

### Bacterial isolates

A total of 704 faecal samples from healthy animals were recovered from different geographic locations of Spain (La Rioja, Aragón, Castilla–La Mancha and Andalucía) during the period from 2013 to 2015. Samples were divided as follows: 178 micromammals (43 wood mouse—*Apodemus sylvaticus*; 2 bank vole—*Clethrionomys glareolus*; 7 greater white-toothed shrew—*Crocidura russula*; 55 common vole—*Microtus arvalis*; 5 western Mediterranean mouse—*Mus spetrus*; 3 brown rat—*Rattus norvegicus*; 2 red squirrel—*Sciurus vulgaris*; 40 black rats—*Rattus rattus*; 1 ferret—*Mustela putorius furo*; 13 house mouse—*Mus musculus*; and other 7 genera); 190 ticks (188 *Ixodes ricinus*, 2 *Dermacentor marginatus*); 124 farmed red deer (*Cervus elaphus*), 65 wild boars (*Sus scrofa*); 51 rabbits (*Oryctolagus cuniculus*); 48 farm animals (40 cows; 5 poultry; 2 pigs and 1 sheep); 34 pets (24 dogs, 3 cats, 4 turtles, 1 coati, 1 fish and 1 parrot) and 14 mallards (*Anas platyrhynchos*). All samples obtained from farmed red deer, wild boars, rabbits, and micromammals were collected in commercial hunting events from hunting areas in Castilla-La Mancha and Andalucía (Spain). Animal handling, including faecal sample collection, was performed or supervised by the Institute for Game and Wildlife Research (IREC, Castilla-La Mancha) according to the regulation of Junta de Comunidades de Castilla-La Mancha. Faecal samples from farm animals, mallards and pets were collected using non-invasive procedures. The ticks were recovered from cows and vegetation, and after external cleaning, they were fully processed. They were divided into 18 groups: 10 groups of female ticks (5–10 ticks/group), 4 groups of males (8–10 ticks/group), one group of 35 larvae, and three groups of 17–20 nymphs/group (*I. ricinus*, n = 188), and two ticks of *D. marginatus*.

Faecal samples and ticks were suspended in saline solution, and enriched in Brain Heart Infusion broth (Becton Dickinson, Rungis, France). After incubation at 37 °C overnight, an aliquot of 200 µL was streaked onto cetrimide agar plates (Becton Dickinson, Rungis, France). Different colonies with *Pseudomonas* morphology were selected per sample (from two to ten colonies), identified by classical biochemical methods (Triple Sugar Iron and oxidase reactions), and confirmed by PCR amplification of 16S rRNA fragment and subsequent sequencing^34^.

### Antimicrobial susceptibility testing

In vitro antimicrobial susceptibility testing was performed by disc diffusion method following the Clinical and Laboratory Standards Institute (CLSI) guidelines^35^. Thirteen antipseudomonal agents were tested: piperacillin, piperacillin–tazobactam, aztreonam, cefepime, ceftazidime, imipenem, meropenem, doripenem, netilmicin, tobramycin, gentamicin, amikacin, and ciprofloxacin. ESBL, MBL and class A carbapenemase phenotypes were studied by double-disc synergic tests^36^.

### Detection and characterization of integrons

The presence of genes encoding type 1, 2 and 3 integrases, 3′-CS of class 1 integrons (*qacE*∆1 + *sul1*) and Tn*402* features was studied by PCR. The characterization of class 1 integron variable regions and gene cassette promoters (Pc) was carried out by PCR mapping and sequencing^34^.

### Molecular typing

The clonal relationship among the recovered isolates was determined by PFGE with *SpeI* restriction enzyme^34^. DNA profiles were analysed by the GelJ software 1.3 (UPGMA algorithm; Dice coefficient)^37^.

MLST for *P. aeruginosa,* was performed by PCR and sequencing^38^. Sequence types (STs) were assigned at the PubMLST database (https://pubmlst.org/paeruginosa/).

### Serotyping

*P. aeruginosa* strains were serotyped by slide agglutination according to the International Antigenic Typing Scheme (IATS) for *P. aeruginosa*, using 16 type O monovalent antisera (O:1 to O:16) (Bio-Rad, Temse, Belgium).

### Detection of virulence markers genes

The presence of the following virulence and quorum sensing genes were analysed by PCR among *P. aeruginosa* strains^39^: *exoU, exoS, exoY, exoT, exoA, exlA, lasA, lasB, aprA, rhlAB, rhlC, rhlI, rhlR, lasI*, and *lasR*. The *exlA* gene was tested with specific designed primers (exlA-F: 5′-TCACCTGGGAAACCTACGAC-3′; exlA-R: 5′-GGCCGCCGGTATAGTAGAAG-3′).

### Biofilm quantification

The analysis of the total biofilm biomass was performed by crystal violet (CV) staining, and the bacterial metabolic activity inside the biofilm structure by fluorescein diacetate (FDA) assay among *P. aeruginosa* isolates. Both methods were carried out by triplicate in flat-bottom microtiter 96-well plates after 24 h of incubation in Müeller–Hinton broth at 37 °C as previously recommended^40^ with some modifications. For CV assay, 66% acetic acid and 10% CV were used, and the FDA working solution concentration was 0.1 mg/mL. Measures were performed using a POLARstar Omega microplate reader (BMG Labtech).

### Elastase and phenazine pigment production

Bacteria were grown overnight in Luria–Bertani (LB) broth at 37 °C with shaking at 120 rpm. After centrifugation, 900 µL of supernatant was used to determine the elastase activity in *P. aeruginosa* isolates by the Elastin Congo Red (ECR) assay as previously described^41^. The chloroform extract method was used to quantify pyocyanin and pyorubin phenazines of *P. aeruginosa* strains by measuring the absorbance of the corresponding solutions at 520 and 525 nm, respectively^42^. All tests were carried out in triplicate.

### Motility

Swarming and swimming motility were studied in *P. aeruginosa* strains. The isolates were grown in LB broth until OD_620nm_ of 0.8 (1 × 10^9^ cells), and then, 4 µL were placed on the middle of 0.5% (swarming) and 0.3% (swimming) LB agar plates. After incubation at 37 °C during 20 h, the plates were imaged with Chemi Doc system (Bio-Rad, Temse, Belgium), and processed with Image Lab software (version 5.2.1, Bio-Rad, Temse, Belgium). The entire plate area was 6,400 mm^2^. All assays were performed in triplicate.

### Whole genome sequencing

The genomic DNA of selected *P. aeruginosa* strains was extracted with the Wizard^®^ Genomic DNA Purification Kit (Promega, Madison, USA). Genomic libraries were performed using TruSeq PCR-free Kit (Illumina, San Diego, USA), and subsequent sequencing was carried out in a HiSeq 1500 (Illumina, San Diego, USA). PLACNETw web tool^43^ was used to genome assembly and to detect mobile genetic elements (MGE), using Velvet as a read assembler^44^ and Prokka for genome annotation and ORF prediction^45^. Description of virulence markers was carried out using the Virulence Factor Database (VFDB) as reference dataset^46^.

The iQTREE^47^ and iTOL^48^ web servers were used to construct a phylogenetic tree comparing our strains with the *exlA*-positive *P. aeruginosa* strains obtained from the *Pseudomonas* Genome Database^15^.

### Cell culture analysis

The A549 epithelial cells and THP-1 monocytes were selected to analyse the strain cytotoxicity. This cytotoxicity was determined by measuring the release of the cytoplasmic enzyme lactate dehydrogenase (LDH) in the culture medium. THP-1 monocytes and A549 epithelial cells were seeded into 96-well plates (at a density of 2.5 × 10^5^ cells/mL for phagocytic cells and of 8 × 10^4^ cells/mL for epithelial cells) and incubated with *P. aeruginosa* strains (10 bacteria/cell) for 4 h. The amount of LDH released was determined using the Cytotoxicity Detection KIT^PLUS^ (LDH) (Roche Diagnostics, Basel, Switzerland). Four independent experiments were performed in quadruplicate.

### Control isolates

*P. aeruginosa* PAO1 strain was included as control in all assays. In the case of the cell culture analysis, *P. aeruginosa* PAO1, *P. aeruginosa* PA7, and *P. aeruginosa* PA14 were used as reference strains.

### Statistical analysis

Statistical analyses of the data were performed using SPSS, program 21.0, using the parametric 1-way ANOVA followed by the Tukey HSD test or two-tailed *t* test (P < 0.05). GraphPad Prism (version 6.01) from GraphPad Software (San Diego, California) was used for graphical representations.

### Ethics approval and consent to participate

Ethics approval was not applicable because this study only involved non-invasive procedures (faecal samples). Animal handling, including faecal sample collection, was performed or supervised by the Institute for Game and Wildlife Research (IREC, Castilla La Mancha) according to local authorities. Animals were not particularly hunted for this study; the IREC group assisted to hunting events for sample collection. Farm animal and pet owners permitted us to study the provided animal faecal samples. Sampling protocols followed all national and international ethical guidelines.

### Disclosures

Part of this study was presented at the 25th European Congress of Clinical Microbiology and Infectious Diseases (ECCMID) (No. 2844, Copenhagen, Denmark, 25–28th April 2015); XXV Congreso Nacional de Microbiología (SEM) (Logroño, Spain, 7–10th July 2015); 26th European Congress of Clinical Microbiology and Infectious Diseases (ECCMID) (No. P1521, Amsterdam, Netherlands, 9–12th April 2016); and 16th International Congress on *Pseudomonas* (No. P68, Liverpool, UK, 5–9th September 2017).

## Supplementary information

Supplementary Information 1.

## Data Availability

All data generated or analysed during this study are included in this published article. A new class 1 integron was submitted to INTEGRALL (https://integrall.bio.ua.pt/) and GenBank databases, and the name In1180 and the accession number KT368820 were assigned. The whole genome data for *Pseudomonas aeruginosa* Ps533, Ps616 and Ps633 have been deposited at GenBank using BioProject number PRJNA526213. The raw sequencing data were deposited at NCBI’s Sequence Read Archive (SRA) under accession numbers SRR8705183 (Ps533), SRR8705184 (Ps616) and SRR8705185 (Ps633), respectively.
