# Supplementary material for: Antimicrobial resistance and virulence of Pseudomonas spp. among healthy animals: concern about exolysin ExlA detection
Source: Sci Rep. 2020 Jul 15;10:11667. doi: 10.1038/s41598-020-68575-1 (PMC7363818; doi:10.1038/s41598-020-68575-1)
Supplement: Supplementary file 1 — Supplementary Information 1. [file 41598_2020_68575_MOESM1_ESM.pdf]

Article Title: Antimicrobial resistance and virulence of *Pseudomonas* spp. among healthy animals: concern about exolysin ExlA detection.

Author names: Lidia Ruiz-Roldán, Beatriz Rojo-Bezares, María de Toro, María López, Paula Toledano, Carmen Lozano, Gabriela Chichón, Lydia Alvarez-Erviti, Carmen Torres, Yolanda Sáenz

Corresponding author:

Yolanda Sáenz
Área de Microbiología Molecular,
Centro de Investigación Biomédica de La Rioja (CIBIR).
C/ Piqueras 98, 3ªplanta, 26006, Logroño, Spain.
Tel.: +34 941278868
Fax: +34 941278887
e-mail: ysaenz@riojasalud.es

Table S1. Number of positive animal samples for *Pseudomonas* spp. isolates.

**Origin (nº of samples)**	**Positive Samples**		**Number of *Pseudomonas* Isolates recovered**
	**Number**	**Proportion (%) per origin / total positive samples**	
Mallard (14)	13	93.0 / 28.3	30
Pets (34)	3	8.8 / 6.5	10
Deer (124)	1	0.8 / 2.2	2
Micromammals (178)	3	1.7 / 6.5	3
Rabbits (51)	3	5.9 / 6.5	5
Ticks (190)	2	1.1 / 4.3	17
Farm animals (48)	2	4.2 / 4.3	9
Wild boars (65)	19	29.2 / 41.3	57
**Total (704)**	**46**	**6.5% / 100%**	**133**

Table S2 Positive samples for *Pseudomonas* spp. isolates and characterisation of those strains.

Origin	Positive Samples (no. of isolates)	Specie (no. of isolates)	Strain selected[a]	Antimicrobial phenotype[b]	PFGE[c]
Mallard	P1 (1)	*P. corrugata*	Ps553	ATM	15
	P2 (1)	*P. koreensis*	Ps566	ATM	23
	P3 (3)	*P. mendocina* (n=2)	Ps542	Susceptible	6
		P. protegens	Ps567	ATM	24
	P4 (3)	*P. fluorescens*	Ps565	ATM	22
		P. plecoglossicida	Ps572	Susceptible	29
		Pseudomonas sp.	Ps577	ATM	33
	P5 (5)	*P. monteillii*	Ps541	ATM	5
		Pseudomonas sp.	Ps552	Susceptible	14
		P. viridiflava	Ps573	ATM	30
		Pseudomonas sp.	Ps576	ATM	32
		Pseudomonas sp.	Ps578	ATM	34
	P6 (1)	*Pseudomonas* sp.	Ps563	ATM	20
	P7 (3)	*P. putida* (n=2)	Ps549	ATM	12
		P. putida	Ps543	ATM	7
	P8 (2)	*P. putida*	Ps548	Susceptible	11
		P. reactans	Ps579	ATM, DOR	35
	P9 (4)	*P. putida*	Ps547	Susceptible	10
		P. protegens	Ps562	Susceptible	19
		P. putida (n=2)	Ps580	ATM	36
	P10 (2)	*P. monteillii*	Ps544	Susceptible	8
		P. pseudoalcaligenes	Ps583	Susceptible	38

	P12 (3)	*P. putida* (n=2)	Ps546	Susceptible	9
		P. putida	Ps571	Susceptible	28
	P13 (1)	*Pseudomonas* sp.	Ps575	ATM	31
	P14 (1)	*P. protegens*	Ps561	ATM	18
Pets	MP21 (1)	*P. koreensis*	Ps538	ATM	4
	MP28 (7)	*P. putida* (n=7)	Ps554	ATM	16
	MP34 (2)	*P. putida* (n=2)	Ps705	ATM	73
Deer	M936/12 (2)	*P. fragi*	Ps550	ATM	13
		P. putida	Ps582	ATM	37
Micromammals	M1385/CIBIR (1)	*P. gessardii*	Ps557	ATM	17
	M1389/CIBIR (1)	*P. koreensis*	Ps569	ATM	26
	M1393/CIBIR (1)	*P. baetica*	Ps570	Susceptible	27
Rabbits	M788/CIBIR (1)	*P. brassicacearum*	Ps584	Susceptible	39
	M794/CIBIR (3)	*P. fulva*	Ps709	MEM, DOR	74
		P. fulva (n=2)	Ps711	MEM, DOR	75
	M795/CIBIR (1)	*P. putida*	Ps568	Susceptible	25
Ticks	1GV15/38 (11)	*P. fluorescens* (n=9)	Ps689[d]	ATM	71
		P. fluorescens (n=2)	Ps693[d]	FEP, ATM	71
	HPAV287 (6)	*P. gessardii* (n=6)	Ps702	FEP, MEM, ATM, DOR*	72
Farm animals	G16 (cow) (1)	*P. cedrina*	Ps564	TZP, FEP, CAZ, IPM, MEM, ATM, DOR*	21
	G48 (sheep) (8)	*Pseudomonas* sp.	Ps535	MEM, ATM	2
		Pseudomonas sp. (n=2)	Ps537	MEM, ATM	3
		P. aeruginosa (n=3)	Ps531	Susceptible	1
		P. aeruginosa (n=2)	Ps533	MEM, ATM	1

Wild boars	M1457/14 (3)	*P. fluorescens*	Ps653	CAZ, ATM, DOR*	58
		P. lundensis	Ps654	Susceptible	59
		P. fluorescens	Ps655	Susceptible	60
	M1458/14 (1)	*Pseudomonas* sp.	Ps643	FEP, ATM	48
	M1459/14 (5)	*P. reactans*	Ps642	ATM, DOR	47
		P. aeruginosa (n=4)	Ps631	Susceptible	42
	M1462/14 (8)	*P. aeruginosa* (n=2)	Ps633	Susceptible	40
		P. aeruginosa	Ps634	DOR	40
		P. psychrophila	Ps644	ATM	49
		P. lundensis	Ps645	Susceptible	50
		P. fragi	Ps646	Susceptible	51
		P. psychrophila	Ps647	FEP, ATM	52
		P. koreensis	Ps648	ATM	53
	M1464/14 (2)	*P. psychrophila*	Ps651	ATM	56
		P. psychrophila	Ps652	Susceptible	57
	M1470/14 (9)	*P. aeruginosa* (n=9)	Ps616	Susceptible	40
	M1471/14 (1)	*Pseudomonas* sp.	Ps639	ATM, DOR	44
	M1472/14 (2)	*P. putida*	Ps649	Susceptible	54
		P. fragi	Ps650	Susceptible	55
	M1474/14 (10)	*P. putida*	Ps640	ATM	45
		P. synxantha	Ps641	Susceptible	46
		P. aeruginosa	Ps620	Susceptible	41
		P. aeruginosa (n=7)	Ps624	Susceptible	40
	M1475/14 (1)	*Pseudomonas* sp.	Ps656[e]	CAZ, ATM	61

M003/15 (2)	*P. fragi*	Ps661	Susceptible	64
	P. fragi	Ps662[f]	Susceptible	65
M004/15 (4)	*P. aeruginosa* (n=4)	Ps638	Susceptible	43
M006/15 (1)	*P. fragi*	Ps663	Susceptible	66
M064/15 (1)	*P. psychrophila*	Ps667	Susceptible	70
M249/15 (1)	*P. trivialis*	Ps664	MEM, ATM	67
M257/15 (3)	*P. fluorescens* (n=3)	Ps658[g]	Susceptible	62
M283/15 (1)	*P. koreensis*	Ps665	ATM	68
M285/15 (1)	*P. fulva*	Ps666	Susceptible	69
M286/15 (1)	*P. psychrophila*	Ps660	Susceptible	63

[a] According to sample, species, PFGE pattern and antimicrobial resistance, these are the 80 *Pseudomonas* strains selected for further characterization in this study.

[b] Susceptible: This strain was susceptible to all 13 antibiotics tested; CAZ, ceftazidime; FEP, cefepime; ATM, aztreonam; IPM, imipenem; MEM, meropenem; DOR, doripenem; TZP, piperacillin-tazobactam.

*These strains showed a multidrug resistance phenotype.

[c] PFGE: Pulsed-field gel electrophoresis.

[d] These strains had a type 1 integrase.

[e] This strain had a class 1 integron In1180 (GenBank accession number KT368820).

[f] This strain had a Tn*402*-like class 1 integron.

[g] This strain had a class 1 integron.

ExlA

```
PA7             MHRDNPVFHLSPRGKLRCLIAGLLLAPHLPQAFAGGLEAAGGPGGTPQLLNQGGVPIVNI    60
Ps616_00602     MHRDNPVFHLSPQGKLRCLIAGLLLAPNLPQAFAGGLEAAGGPGGTPQLLNQGGVPIVNI    60
Ps633_03533     MHRDNPVFHLSPQGKLRCLIAGLLLAPNLPQAFAGGLEAAGGPGGTPQLLNQGGVPIVNI    60
Ps533_00091     MHRDNPVFHLSPQGKLRCLIAGLLLAPNLPQAFAGGLEAAGGPGGTPQLLNQGGVPIVNI    60
CF_PA39         MHRDNPVFHLSPQGKLRCLIAGLLLAPNLPQAFAGGLEAAGGPGGTPQLLNQGGVPIVNI    60
                ************:**************:********************************

PA7             VAPNAAGLSHNQFLDYNVDRQGLVLNNALQAGASQLAGQLAANPQLRGDAASAILNEVIS    120
Ps616_00602     VAPNAAGLSHNQFLDYNVDRQGLVLNNALQSGTSQLAGQLAANPQLRGDAASAILNEVIS    120
Ps633_03533     VAPNAAGLSHNQFLDYNVDRQGLVLNNALQSGTSQLAGQLAANPQLRGDAASAILNEVIS    120
Ps533_00091     VAPNAAGLSHNQFLDYNVDRQGLVLNNALQSGTSQLAGQLAANPQLRGDAASAILNEVIS    120
CF_PA39         VAPNAAGLSHNQFLDYNVDRQGLVLNNALQSGTSQLAGQLAANPQLRGDAASAILNEVIS    120
                ******************************:*:***************************

PA7             RNASAINGPQEIFGQAADYILANPNGISVNGGSFINTPHASLLVGRPELADGKLQALNTN    180
Ps616_00602     RNASAINGPQEIFGQAADYILANPNGISVNGGSFINTPQASLLVGRPELADGKLQALNTG    180
Ps633_03533     RNASAINGPQEIFGQAADYILANPNGISVNGGSFINTPQASLLVGRPELADGKLQALNTG    180
Ps533_00091     RNASAINGPQEIFGQAADYILANPNGISVNGGSFINTPQASLLVGRPELADGKLQALNTG    180
CF_PA39         RNASAINGPQEIFGQAADYILANPNGISVNGGSFINTPQASLLVGRPELADGKLQALNTG    180
                **************************************:********************.

PA7             DAVGALQIQGQGLSNRDGSIALLAPRVDSQGKIEASAELDLTVGRNRIDYPSGKVERDPS    240
Ps616_00602     DAVGALQIHDQGLSNRGGSIALLAPRVDSQGKIEASTELDLTVGRNRIDYPSGKIERAPS    240
Ps633_03533     DAVGALQIHDQGLSNRGGSIALLAPRVDSQGKIEASTELDLTVGRNRIDYPSGKIERAPS    240
Ps533_00091     DAVGALQIHDQGLSNRGGSIALLAPRVDSQGKIEASTELDLTVGRNRIDYPSGKIERAPS    240
CF_PA39         DAVGALQIHDQGLSNRGGSIALLAPRVDSQGKIEASTELDLTVGRNRIDYPSGKIERAPS    240
                ********:.******.*******************:*****************:** **

PA7             GDVRPGERRIDASLFGAMQAGRINILSTAEGAGVRVGPVGIDGRDGVDLRSAGDLSISGQ    300
Ps616_00602     GDLRPGERRIDASLFGAMQAGRINILSTAEGAGVRIGPVGIDGKDGVDLRSAGDLSISGQ    300
Ps633_03533     GDLRPGERRIDASLFGAMQAGRINILSTAEGAGVRIGPVGIDGKDGVDLRSAGDLSISGQ    300
Ps533_00091     GDLRPGERRIDASLFGAMQAGRINILSTAEGAGVRIGPVGIDGKDGVDLRSAGDLSISGQ    300
CF_PA39         GDLRPGERRIDASLFGAMQAGRINILSTAEGAGVRIGPVGIDGKDGVDLRSAGDLSISGQ    300
                **:********************************:*******:****************

PA7             ALPDNSLNALRAAIRSDSGNVGLHARGDLSLAADVSGGRVDLKSGRNLTLGSVESRNLR    360
Ps616_00602     ALPDNSLNALRAAIRSDSGNVGLHARGDLNLAADVSGGRVDLKSGRNLTLDSVESRNLR    360
Ps633_03533     ALPDNSLNALRAAIRSDSGNVGLHARGDLNLAADVSGGRVDLKSGRNLTLDSVESRNLR    360
Ps533_00091     ALPDNSLNALRAAIRSDSGNVGLHARGDLNLAADVSGGRVDLKSGRNLTLDSVESRNLR    360
CF_PA39         ALPDNSLNALRAAIRSDSGNVGLHARGDLNLAADVSGGRVDLKSGRNLTLDSVESRNLR    360
                *****************************.********************.********

PA7             ESRERWSNSTIGITWETYDRTRTVTDSKQHGSRIDARADASLAARGDSELRAATVKAGAT    420
Ps616_00602     ESRERWSNSTIGITWETYDRTRTVTDSKQHGSRIDARADASLAARGDSELRAATVKTGAT    420
Ps633_03533     ESRERWSNSTIGITWETYDRTRTVTDSKQHGSRIDARADASLAARGDSELRAATVKTGAT    420
Ps533_00091     ESRERWSNSTIGITWETYDRTRTVTDSKQHGSRIDARADASLAARGDSELRAATVKTGAT    420
CF_PA39         ESRERWSNSTIGITWETYDRTRTVTDSKQHGSRIDARADASLAARGDSELRAATVKTGAT    420
                ********************************************************:***

PA7             LKVSSGGDTRLLAATETRTERDQGAHRKHLWKANWDKGSSEQRSVASSLEGARVELGGGR    480
Ps616_00602     LKVSSGGDTRLLAATETRTERDQGAHRKHLWKANWDKGSSEQRSVASSLEGARVELGGGR    480
Ps633_03533     LKVSSGGDTRLLAATETRTERDQGAHRKHLWKANWDKGSSEQRSVASSLEGARVELGGGR    480
Ps533_00091     LKVSSGGDTRLLAATETRTERDQGAHRKHLWKANWDKGSSEQRSVASSLEGARVELGGGR    480
CF_PA39         LKVSSGGDTRLLAATETRTERDQGAHRKHLWKANWDKGSSEQRSVASSLEGARVELGGGR    480
                ************************************************************

PA7             RLNLEGADVASRGDLDLQAKSVDIGSASRSHSSRDNSYSGDLVGGSFFGSHGDGDSGKTL    540
Ps616_00602     RLNLEGAEVASRGDLDLQAKSVDIGSASRSHNSRDNSYSGDLVGGSFFGRHGNGDSGQTL    540
Ps633_03533     RLNLEGAEVASRGDLDLQAKSVDIGSASRSHNSRDNSYSGDLVGGSFFGRHGNGDSGQTL    540
Ps533_00091     RLNLEGAEVASRGDLDLQAKSVDIGSASRSHNSRDNSYSGDLVGGSFFGRHDDGDSGQTL    540
CF_PA39         RLNLEGAEVASRGDLDLQAKSVDIGSASRSHNSRDNSYSGDLVGGSFFGRHDDGDSGQTL    540
                *******:***********************.***************** *.:****:**
```

```
PA7            QQGSRVKADGALTVTADAVEVRGSQVRGARKAEVVSGKGSLRIDGVEETAHSNSYSKDSK   600
Ps616_00602    QQGSRVKADGALTVTADAVEVRGSQVRGARKAEVVSGKGSLRIDGEEETAHSNSYSKDSK   600
Ps633_03533    QQGSRVKADGALTVTADAVEVRGSQVRGARKAEVVSGKGSLRIDGEEETAHSNSYSKDSK   600
Ps533_00091    QQGSRVKADGALTVTADAVEVRGSQVRGARKAEVVSGKGSLRIDGEEETAHSNSYSKDSK   600
CF_PA39        QQGSRVKADGALTVTADAVEVRGSQVRGARKAEVVSGKGSLRIDGEEETAHSNSYSKDSK   600
               ********************************************* **************

PA7            FFGIAKDESRQRSKDSSNRASEVRSDSNLTLRSAADIAIRGSRVEAGGALAAEAKGNLEI   660
Ps616_00602    FFGIAKEESRQRSKDSSNRASEVRSDSNLTLRSAAGIAIRGSRVEAGGALAAEAKGNLEI   660
Ps633_03533    FFGIAKEESRQRSKDSSNRASEVRSDSNLTLRSAAGIAIRGSRVEAGGALAAEAKGNLEI   660
Ps533_00091    FFGIAKEESRQRSKDSSNRASEVRSDSNLTLRSAAGIAIRGSRVEAGGALAAEAKGNLEI   660
CF_PA39        FFGIAKEESRQRSKDSSNRASEVRSDSNLTLRSAAGIAIRGSRVEAGGALAAEAKGNLEI   660
               ******:****************************.************************

PA7            ASAQERHDGNDSRHTRGFDAYAGEQTPGSRQYRAGVRYQDQRTSVRREETRNSGSSLGGA   720
Ps616_00602    ASAQERYDGSDSRHTRGFGAYAGEQTPGSRQYRAGVRYQDQRTSVRREETHNSGASLGGA   720
Ps633_03533    ASAQERYDGSDSRHTRGFGAYAGEQTPGSRQYRAGVRYQDQRTSVRREETHNSGASLGGA   720
Ps533_00091    ASAQERYDGSDSRHTRGFGAYAGEQTPGSRQYRAGVRYQDQRTSVRREETHNSGASLGGA   720
CF_PA39        ASAQERYDGSDSRHTRGFGAYAGEQTPGSRQYRAGVRYQDQRTSVRREETHNSGASLGGA   720
               ******:**.********.*******************************:***:*****

PA7            SLAVKAGGDLTVKGAELKASAGDASLSGKNVALLAEQDSKTRSSEQTTTGGGFYYTGGLD   780
Ps616_00602    SLAVKAGGDLTVKGAELKASAGDASLSGKNVALLAEQDGKTRSREQTTTGGGFYYTGGLD   780
Ps633_03533    SLAVKAGGDLTVKGAELKASAGDASLSGKNVALLAEQDGKTRSREQTTTGGGFYYTGGLD   780
Ps533_00091    SLAVKAGGDLTVKGAELKASAGDASLSGKNVALLAEQDGKTRSREQTTTGGGFYYTGGLD   780
CF_PA39        SLAVKAGGDLTVKGAELKASAGDASLSGKNVALLAEQDGKTRSREQTTTGGGFYYTGGLD   780
               **************************************.**** ****************

PA7            RAGSGIEVGHQRIDENDAESHARTSQVNATGNLRIDAAQGSLTTQGARLEAGDSLAVAAG   840
Ps616_00602    RAGSGIEVGHQRIDENDAESHARTSQVNATGNLRIDAARGSLTTQGARLEAGDSLAVAAG   840
Ps633_03533    RAGSGIEVGHQRIDENDAESHARTSQVNATGNLRIDAARGSLTTQGARLEAGDSLAVAAG   840
Ps533_00091    RAGSGIEVGHQRIDENAAESHARTSQVNATGNLRIDAARGSLTTQGARLEAGDSLAVAAG   840
CF_PA39        RAGSGIEVGHQRIDENAAESHARTSQVNATGNLRIDAARGSLTTQGARLEAGDSLAVAAG   840
               **************** *********************:*********************

PA7            TVDNQAARDSQSSQRHDSGWSGDIGANLEYRGIARPIEKAVEGVAQRKVHQPGLLDNLEQ   900
Ps616_00602    AVDNQAARDSRSSRRHDNSWSGDVGANLEYRGIARPIEKAVEGVAQRKVHQPGLLDNLEQ   900
Ps633_03533    AVDNQAARDSRSSRRHDNSWSGDVGANLEYRGIARPIEKAVEGVAQRKVHQPGLLDNLEQ   900
Ps533_00091    AVDNQAARDSRSSRRHDNSWSGDVGANLEYRGIARPIKKAVEGVAQRKVHQPGLLDNLEQ   900
CF_PA39        AVDNQAARDSRSSRRHDNSWSGDVGANLEYRGIARPIEKAVEGVAQRKVHQPGLLDNLEQ   900
               :*********:**:***..****:*************:**********************

PA7            PNVGVDLEISHRDSRGEQQASQAQVSSFAGGQVELKVGDALRDEGTRYQARSGGLLIDAA   960
Ps616_00602    PNVGVDLEISHRDSRGEQQASQAQVSSFAGGQVDLKVGDALRDEGTRYQARSGGLLIDAA   960
Ps633_03533    PNVGVDLEISHRDSRGEQQASQAQVSSFAGGQVDLKVGDALRDEGTRYQARSGGLLIDAA   960
Ps533_00091    PNVGVDLEISHRDSRGEQQASQAQVSSFAGGQVDLKVGDALRDEGTRYQARSGGLLIDAA   960
CF_PA39        PNVGVDLEISHRDSRGEQQASQAQVSSFAGGQVDLKVGDALRDEGTRYQARSGGLLIDAA   960
               *********************************:**************************

PA7            RHDARAAENTSGSHEQSLDAKVGGRLYTTTGQDLNLRLSGIGGSSENSASQTTAVVGEYA   1020
Ps616_00602    RHDARTSENTSGSHEQSLDAKAGGRLYTTTGQDLNLRLSGNGGSSENSASQTTAVVGEYA   1020
Ps633_03533    RHDARTSENTSGSHEQSLDAKAGGRLYTTTGQDLNLRLSGNGGSSENSASQTTAVVGEYA   1020
Ps533_00091    RHDARASENTSGSHEQSLDAKAGGRLYTTTGQDLNLRLSGNGGSSENSASQTTAVVGEYA   1020
CF_PA39        RHDARASENTSGSHEQSLDAKAGGRLYTTTGQDLNLRLSGNGGSSENSASQTTAVVGEYA   1020
               *****::**************.****************** *******************

PA7            AKQGVEIRLGGDGLYQGSRFDGGEAGVRLSAGGNLALEQANDRQSASSASLRGDAALSGG   1080
Ps616_00602    AKQGVEIRLGGDGLYRGGRFDGGKGGVRLSAGGNLALEQANDRQGASSASLRGDAALSGG   1080
Ps633_03533    AKQGVEIRLGGDGLYRGGRFDGGKGGVRLSAGGNLALEQANDRQGASSASLRGDAALSGG   1080
Ps533_00091    AKQGVEIRLGGDGLYRGGRFDGGKGGVRLSAGGNLALEQANDRQGASSASLRGDAALSGG   1080
CF_PA39        AKQGVEIRLGGDGLYRGGRFDGGKGGVRLSAGGNLALEQANDRQGASSASLRGDAALSGG   1080
               ***************:*.*****:.*******************.***************
```

```
PA7            MAPSANGKGLNASAGLQLDHKAGDSRDSQARVADIQAKGAVELRSGGDLVLQGSNIGSAA   1140
Ps616_00602    MAPSAHGKGLNASAGLQLDHKARDSRDSQARVADIRAKGTVELRSGGDLVLQGSNIGSAA   1140
Ps633_03533    MAPSAHGKGLNASAGLQLDHKARDSRDSQARVADIRAKGTVELRSGGDLVLQGSNIGSAA   1140
Ps533_00091    MAPSAHGKGLNASAGLQLDHKARDSRDSQARVADIRAKGTVELRSGGDLVLQGSNIGSAA   1140
CF_PA39        MAPSAHGKGLNASAGLQLDHKARDSRDSQARVADIRAKGTVELRSGGDLVLQGSNIGSAA   1140
               *****:**************** ************:***:********************

PA7            AKTGDIVLAAGGKLDLQAARDSHRAGGNNLGGGFSLGGGSVRDAETSSKNGSVSGNFNIG   1200
Ps616_00602    AKTGDIVLAAGGKLDLRAARDSHQARGDNLGGGFTLGGSSVRSAETSSRNGSVSGNFNIG   1200
Ps633_03533    AKTGDIVLAAGGKLDLRAARDSHQARGDNLGGGFTLGGSSVRSAETSSRNGSVSGNFNIG   1200
Ps533_00091    AKTGDIVLAAGGKLDLRAARDSHQARGDNLGGGFTLGGSSVRSAETSSRNGSVSGNFNIG   1200
CF_PA39        AKTGDIVLAAGGKLDLRAARDSHQARGDNLGGGFTLGGSSVRSAETSSRNGSVSGNFNIG   1200
               ****************:******:* *:******:***.***.*****:***********

PA7            RVDEERHALNGGNLHSATKASLSSAADDATAVRLQGTRIEAAQVSLEAGNGGILQESAES   1260
Ps616_00602    RVDEQRHALNGGNLHSATKASLSSAADDATAVRLQGTRIEAARVSLEAGNGGILQESAES   1260
Ps633_03533    RVDEQRHALNGGNLHSATKASLSSAADDATAVRLQGTRIEAARVSLEAGNGGILQESAES   1260
Ps533_00091    RVDEQRHALNGGNLHSATKASLSSAADDATAVRLQGTRIEAARVSLEAGNGGILQESAES   1260
CF_PA39        RVDEQRHALNGGNLHSATKASLSSAADDATAVRLQGTRIEAARVSLEAGNGGILQESAES   1260
               ****:*************************************:*****************

PA7            SERRDNWGVLLGAGANGGKTTGAPSDYRSDYAVQARAKVDVDVLRSQTQGDSVIQADRVI   1320
Ps616_00602    GERRDNWGVLLGAGVNGGKTTGAPSDYRTNHAVQARAKVDVDVLRSQTQGDSVIQADQVT   1320
Ps633_03533    GERRDNWGVLLGAGVNGGKTTGAPSDYRTNHAVQARAKVDVDVLRSQTQGDSVIQADQVT   1320
Ps533_00091    GERRDNWGVLLGAGVNGGKTTGAPSDYRTNHAVQARAKVDVDVLRSQTQGDSVIQADQVT   1320
CF_PA39        GERRDNWGVLLGAGVNGGKTTGAPSDYRTNHAVQARAKVDVDVLRSQTQGDSVIQADQVT   1320
               .*************.*************:::**************************:*

PA7            LASQGDTRLEGARIDAAQVDGRIGGDLRVESRQDRAEGVKVNVDARLGVEKNQPGLVNKL   1380
Ps616_00602    LASQGDTRLEGARIDAARVDGRIGGDLRVESRQDRAEGVKVNVDARLGVEKNQPGLVNKL   1380
Ps633_03533    LASQGDTRLEGARIDAARVDGRIGGDLRVESRQDRAEGVKVNVDARLGVEKNQPGLVNKL   1380
Ps533_00091    LASQGDTRLEGARIDAARVDGRIGGDLRVESRQDRAEGVKVNVDARLGVEKNQPGLVNKL   1380
CF_PA39        LASQGDTRLEGARIDAARVDGRIGGDLRVESRQDRAEGVKVNVDARLGVEKNQPGLVNKL   1380
               *****************:******************************************

PA7            ASKTGPLKDKLETKAENAFDKHRGKLENGIDRNVERLGKAGDNLLAKAEKAKERLGEKLV   1440
Ps616_00602    ASKTGPLKDKLETKAENAFDKHRGKLENGIDRNVERLGKAGDNLLAKAETAKERLGEKRV   1440
Ps633_03533    ASKTGPLKDKLETKAENAFDKHRGKLENGIDRNVERLGKAGDNLLAKAETAKERLGEKRV   1440
Ps533_00091    ASKTGPLKDKLETKAENAFDKHRGKLENGIDRNVERLGKAGDNLLAKAETAKERLGEKRV   1440
CF_PA39        ASKTGPLKDKLETKAENAFDKHRGKLENGIDRNVERLGKAGDNLLAKAETAKERLGEKRV   1440
               *************************************************.******** *

PA7            RSGSYEVNPEPRGAFASKLDRARGYLAEKGEALGDRLSGLKQRLSPNKTGSYVVNDKQTA   1500
Ps616_00602    RSGSYEVNPEPRGAFASKLDRARGYLAEKGEALGDRLSGLKQRLSPNKTGSYAVNDRQTT   1500
Ps633_03533    RSGSYEVNPEPRGAFASKLDRARGYLAEKGEALGDRLSGLKQRLSPNKTGSYAVNDRQTT   1500
Ps533_00091    RSGSYEVNPEPRGAFASKLDRARGYLAEKGEALGDRLSGLKQRLSPNKTGSYAVNDRQTT   1500
CF_PA39        RSGSYEVNPEPRGAFASKLDRARGYLAEKGEALGDRLSGLKQRLSPNKTGSYAVNDRQTT   1500
               ****************************************************.***:**:

PA7            GAKVGNAAENVLFGDKSGEASVTPTLYLDVSHVSRNYVTEASGITGRQGVNLQVGAATQL   1560
Ps616_00602    GAKVGNAAESVLFGDKSGEASVTPTLYLDVSHVSRNYVTEASGITSRQGVTLQVGAATRL   1560
Ps633_03533    GAKVGNAAESVLFGDKSGEASVTPTLYLDVSHVSRNYVTEASGITSRQGVTLQVGAATRL   1560
Ps533_00091    GAKVGNAAESVLFGDKSGEASVTPTLYLDVSHVSRNYVTEASGITSRQGVTLQVGAATRL   1560
CF_PA39        GAKVGNAAESVLFGDKSGEASVTPTLYLDVSHVSRNYVTEASGITGRQGVTLQVGAATRL   1560
               *********.***********************************.****.*******:*

PA7            TGARISASDGKVDLGGSRVETRALAGKDYRADLGLNVSRSPVDLAFGIKDEFSQEHDQAT   1620
Ps616_00602    TGARISASDGKVALGGSRVETHVLAGKDYRADLGLNLSRSPVDLALGIKDEFSQEHDQAT   1620
Ps633_03533    TGARISASDGKVALGGSRVETHVLAGKDYRADLGLNLSRSPVDLALGIKDEFSQEHDQAT   1620
Ps533_00091    TGARISASDGKVALGGSRVETHVLAGKDYRADLGLNLSRSPVDLALGIKDEFSQEHDQAT   1620
CF_PA39        TGARISASDGKVALGGSRVETHVLAGKDYRADLGLNLSRSPVDLALGIKDEFSQEHDQAT   1620
               ************ ********:.*************:********:**************
```

```
PA7             RDDQAFNLGALRVGGRNRDQQLQAGIEQKAD   1651
Ps616_00602     RDDQAFNLGALRVGGRNRDQQLQAGIEQKAD   1651
Ps633_03533     RDDQAFNLGALRVGGRNRDQQLQAGIEQKAD   1651
Ps533_00091     RDDQAFNLGALRVGGRNRDQQLQAGIEQKAD   1651
CF_PA39         RDDQAFNLGALRVGGRNRDQQLQAGIEQKAD   1651
                *******************************
```

ExlB

```
PA7             MPYRSTESSPRRPFCRALLLGLLGASPVFAADPGQPGQEALRQHQQQQLELQRMQLEERQ   60
Ps533_00090     MPYRSTESSPRRPFCRALLLGLLGASPAFAADPGQPGQEALRQHQQQQLELQRMQLEERQ   60
Ps616_00603     MPYRSTESSPRRPFCRALLLGLLGASPAFAADPGQPGQEALRQHQQQQLELQRMQLEERQ   60
Ps633_03532     MPYRSTESSPRRPFCRALLLGLLGASPAFAADPGQPGQEALRQHQQQQLELQRMQLEERQ   60
CF_PA39         MPYRSTESSPRRPFCRALLLGLLGASPAFAADPGQPGQEALRQHQQQQLELQRMQLEERQ   60
                ***************************.********************************

PA7             RQLQRGNFGGSTAPAPASPPIADHDCWPLSGTRIGGVTLLSRERLDRTLGPLIGECMGPG   120
Ps533_00090     RQLQRGNFGGSTAPAPVSPPIADHDCWPLSGTRVGGVTLLSRGRLDRTLGPLISECMGPG   120
Ps616_00603     RQLQRGNFGGSTAPAPVSPPIADHDCWPLSGTRVGGVTLLSRGRLDRTLGPLISECMGPG   120
Ps633_03532     RQLQRGNFGGSTAPAPVSPPIADHDCWPLSGTRVGGVTLLSRGRLDRTLGPLISECMGPG   120
CF_PA39         RQLQRGNFGGSTAPAPVSPPIADHDCWPLSGTRVGGVTLLSRGRLDRTLGPLISECMGPG   120
                ****************.****************:******** **********.******

PA7             QINRLLAAITRLYVDAGYVAARPYLANPPEAGQSLDILVDEGYVESIELDDQSLPVSLRG   180
Ps533_00090     QINRLLAAITHLYVDAGYVAARPYLANPPEAGQSLDILVDEGYVESIELDDQGLPVSLRG   180
Ps616_00603     QINRLLAAITHLYVDAGYVAARPYLANPPEAGQSLDILVDEGYVESIELDDQSLPVSLRG   180
Ps633_03532     QINRLLAAITHLYVDAGYVAARPYLANPPEAGQSLDILVDEGYVESIELDDQSLPVSLRG   180
CF_PA39         QINRLLAAITHLYVDAGYVAARPYLANPPEAGQSLDILVDEGYVESIELDDQGLPVSLRG   180
                **********:*****************************************.*******

PA7             AFPGVLGKPLNLRDLEQGLDQLNRLRSVDLAADIAPGSQPGASRILLRKRGGAARWSLGL   240
Ps533_00090     AFPGMLGKPLNLRDLEQGLDQLNRLRSVDLAADIAPGSQPGASRILLRKRGGAARWSLGL   240
Ps616_00603     AFPGMLGKPLNLRDLEQGLDQLNRLRSVDLAADIAPGSQPGASRILLRKRGGAARWSLGL   240
Ps633_03532     AFPGMLGKPLNLRDLEQGLDQLNRLRSVDLAADIAPGSQPGASRILLRKRGGAARWSLGL   240
CF_PA39         AFPDMLGKPLNLRDLEQGLDQLNRLRSVDLAADIAPGSQPGASRILLRKRGGAARWSLGL   240
                ***.:*******************************************************

PA7             GLDNLGSLATGRDRGNLSLGLDSPLELNDSLLLSASDTLNRDRRYSRSSSLYYAIPYGYW   300
Ps533_00090     GLDNLGSLATGRDRGNLSLGLDSPLELNDSLLLSASDTLNRDRRYSRSSSLYYAIPYGYW   300
Ps616_00603     GLDNLGSLATGRDRGNLSLGLDSPLELNDSLLLSASDTPNRDRRYSRSSSLYYAIPYGYW   300
Ps633_03532     GLDNLGSLATGRDRGNLSLGLDSPLELNDSLLLSASDTPNRDRRYSRSSSLYYAIPYGYW   300
CF_PA39         GLDNLGSLATGRDRGNLSLGLDSPLELNDSLLLSASDTPNRDRRYSRSSSLYYAIPYGYW   300
                ************************************** *********************

PA7             TYSLFASHAEFRSPLRTGRQTFYQTGATDLFSLRADRVLWRGQRHQLSANLQLARKDVDS   360
Ps533_00090     TYSLFVSHAEFRSPLRTGRQTFYQTGATDLFSLRADRVLWRGQRHQLSANLQLARKDVDS   360
Ps616_00603     TYSLFASHAEFRSPLRTGRQTFYQTGATDLFSLRADRVLWRGQRHQLSANLQLARKDVDS   360
Ps633_03532     TYSLFASHAEFRSPLRTGRQTFYQTGATDLFSLRADRVLWRGQRHQLSANLQLARKDVDS   360
CF_PA39         TYSLFASHAEFRSPLRTGRQTFYQTGATDLFSLRADRVLWRGQRHQLSANLQLARKDVDS   360
                *****.******************************************************

PA7             YLGGIRLTNQSPRLSVVEAGLNLFWLDSAVWSLDLGYARGLSALGADRDRAGRADNYPRA   420
Ps533_00090     YLGGIRLSNQSPRLSVVEAGLNLFWLDSAVWSLDLGYARGLSALGADRDRPGRADNYPRA   420
Ps616_00603     YLGGIRLSNQSPRLSVVEAGLNLFWLDSAVWSLDLGYARGLSALGADRDRPGRADNYPRA   420
Ps633_03532     YLGGIRLSNQSPRLSVVEAGLNLFWLDSAVWSLDLGYARGLSALGADRDRPGRADNYPRA   420
CF_PA39         YLGGIRLSNQSPRLSVVEAGLNLFGLDSAVWSLDLGYARGLSALGADRDRPGRADNYPRA   420
                *******:**************** ************************* *********

PA7             QFDKYRLGLNQWRNGQLAGQAWQWQSQLTAQYSADPLPAIEQLLATDDSAVRGFRRNSVS   480
Ps533_00090     QFDKYRLGLNQWRNGQLAGQAWQWQSQFTAQYSTDPLPAIEQLLATDDSAVRGFRRNSVS   480
Ps616_00603     QFDKYRLGLNQWRNGQLAGQAWQWQSQFTAQYSTDPLPAIEQLLATDDSAVRGFRRNSVS   480
Ps633_03532     QFDKYRLGLNQWRNGQLAGQAWQWQSQFTAQYSTDPLPAIEQLLATDDSAVRGFRRNSVS   480
CF_PA39         QFDKYRLGLNQWRNGQLAGQAWQWQSQFTAQYSTDPLPAIEQLLATDDSAVRGFRRNSVS   480
                ***************************:*****:**************************
```

```
PA7             GAIGAIWRNTLRLPLRSDLPVQVTPRLGLDHGWVKLYHGAPGQYLSGASAGLNLSWKQVQ         540
Ps533_00090     GAIGAVWRNTLRLPLRSDLPVQVTPRLGLDHGWVKLYHGVPGQYLSGASAGLNLSWKQVQ         540
Ps616_00603     GAIGAVWRNTLRLPLRSDLPVQVTPRLGLDHGWVKLYHGVPGQYLSGASAGLNLSWKQVQ         540
Ps633_03532     GAIGAVWRNTLRLPLRSDLPVQVTPRLGLDHGWVKLYHGVPGQYLSGASAGLNLSWKQVQ         540
CF_PA39         GAIGAVWRNTLRLPLRSDLPVQVTPRLGLDHGWVKLYHGVPGQYLSGASAGLNLSWKQVQ         540
                *****:*********************************.********************

PA7             LDLDYQRSLSAPSAFRHEPESWLLRLSLQI    570
Ps533_00090     LDLDYQRSLSAPAAFRHEPESWLLRLSLQI    570
Ps616_00603     LDLDYQRSLSAPAAFRHEPESWLLRLSLQI    570
Ps633_03532     LDLDYQRSLSAPAAFRHEPESWLLRLSLQI    570
CF_PA39         LDLDYQRSLSAPAAFRHEPESWLLRLSLQI    570
                ************:*****************
```

Figure S1 ClustalW2 alignments of ExlA and ExlB proteins of the three *P. aeruginosa* strains of this study, *P. aeruginosa* PA7 reference strain (PSPA7_4642 – PSPA7_4641) and *P. aeruginosa* CF_PA39 (AX20_RS0121405 - AX20_RS0121410). The protein sequences of reference strains were obtained from The Pseudomonas Genome Database [15].

Amino acid changes comparing with the PA7 reference strain are highlighted in green. Conserved motifs involved in TPS secretion are coloured in purple. The RGD motifs of ExlA are in bold red letters.

Table S3 Comparison of amino acid substitutions of ExlA and ExlB proteins of the three *P. aeruginosa* strains of this study, *P. aeruginosa* PA7 reference strain (PSPA7_4642 - PSPA7_4641) and *P. aeruginosa* CF_PA39 (AX20_RS0121405 - AX20_RS0121410). The protein sequences of reference strains were obtained from The Pseudomonas Genome Database [15].

**ExlA PA7 (amino acid and position)**	**CF_PA39**	**Ps533**	**Ps616**	**Ps633**
R13	Q	Q	Q	Q
H28	N	N	N	N
A91	S	S	S	S
A93	T	T	T	T
H159	Q	Q	Q	Q
N180	G	G	G	G
Q189	H	H	H	H
G190	D	D	D	D
D197	G	G	G	G
A217	T	T	T	T
V235	I	I	I	I
D238	A	A	A	A
V243	L	L	L	L
V276	I	I	I	I
R284	K	K	K	K
S330	N	N	N	N
G352	D	D	D	D
A417	T	T	T	T
D488	E	E	E	E
S512	N	N	N	N
S530	R	R	R	R
G532	D	D	-	-
D533	-	-	N	N
K538	Q	Q	Q	Q

V586	E	E	E	E
D607	E	E	E	E
D636	G	G	G	G
H667	Y	Y	Y	Y
N670	S	S	S	S
D679	G	G	G	G
R711	H	H	H	H
S715	A	A	A	A
S759	G	G	G	G
S764	R	R	R	R
D797	A	A	-	-
Q819	R	R	R	R
T841	A	A	A	A
Q851	R	R	R	R
Q854	R	R	R	R
S858	N	N	N	N
G859	S	S	S	S
I864	V	V	V	V
E878	-	K	-	-
E934	D	D	D	D
A966	-	-	T	T
A967	S	S	S	S
V982	A	A	A	A
I1001	N	N	N	N
Q1036	R	R	R	R
S1038	G	G	G	G
E1044	K	K	K	K
A1045	G	G	G	G
S1065	G	G	G	G
N1086	H	H	H	H
G1103	R	R	R	R
Q1116	R	R	R	R
A1120	T	T	T	T
Q1157	R	R	R	R

R1164	Q	Q	Q	Q
G1166	R	R	R	R
N1168	D	D	D	D
S1175	T	T	T	T
G1179	S	S	S	S
D1183	S	S	S	S
K1189	R	R	R	R
E1205	Q	Q	Q	Q
Q1243	R	R	R	R
S1261	G	G	G	G
A1275	V	V	V	V
S1289	T	T	T	T
D1290	N	N	N	N
Y1291	H	H	H	H
R1318	Q	Q	Q	Q
I1320	T	T	T	T
Q1338	R	R	R	R
K1430	T	T	T	T
L1439	R	R	R	R
V1493	A	A	A	A
K1497	R	R	R	R
A1500	T	T	T	T
N1510	S	S	S	S
G1546	-	-	S	S
N1551	T	T	T	T
Q1559	R	R	R	R
D1573	A	A	A	A
R1582	H	H	H	H
A1583	V	V	V	V
V1597	L	L	L	L
F1606	L	L	L	L

ExlB PA7 (amino acid and position)	CF_PA39	Ps533	Ps616	Ps633
V28	A	A	A	A
A77	V	V	V	V
I94	V	V	V	V
E103	G	G	G	G
G114	S	S	S	S
R131	H	H	H	H
S173	G	G	-	-
G184	D	-	-	-
V185	M	M	M	M
L279	P	-	P	P
A306	-	V	-	-
T368	S	S	S	S
W385	T	-	-	-
A411	P	P	P	P
L448	F	F	F	F
A454	T	T	T	T
I486	V	V	V	V
A520	V	V	V	V
S553	A	A	A	A

- No differences in comparison with PA7 amino acid.
